# Feeling Supported, Perceived Usefulness, and Psychological Distress Among Adults in Japan: A Cross-Sectional Study

**DOI:** 10.3390/healthcare14203364

**Published:** 2026-10-09

**Authors:** Shimpei Hayashi, Shigeaki Masuda

**Affiliations:** Department of Practical Fundamental Nursing, Faculty of Nursing, School of Medicine, Nara Medical University, Kashihara 634-8521, Japan; masuda@naramed-u.ac.jp

**Keywords:** psychological distress, feeling supported, perceived usefulness, social support, social roles, cross-sectional study, Japan

## Abstract

**Background/Objectives:** Feeling supported and perceiving oneself as useful to others may be associated with psychological distress. We examined these associations and exploratory nonlinear and interaction patterns in a general-population internet sample in Japan. **Methods:** This cross-sectional secondary analysis included 10,199 respondents aged ≥20 years from the Survey on Satisfaction and Quality of Life, 2025. The two social perceptions were single items scored 0–10. A six-item K6-derived distress score (0–24) used a modified reference period. Covariate-adjusted ordinary least squares regression included centered linear, quadratic, and product terms, with HC3 robust inference. **Results:** Near the centering values, feeling supported (B = −0.552, 95% CI −0.620 to −0.484) and perceived usefulness (B = −0.744, 95% CI −0.816 to −0.673) were inversely associated with distress. Both quadratic terms were positive in the primary model. The negative interaction (B = −0.050, 95% CI −0.081 to −0.019) added only 0.0011 to R^2^. Its magnitude varied with range restriction and additional covariates; log-transformed and spline analyses also indicated a joint association. The age group modification estimate was imprecise (B = 0.059, 95% CI −0.023 to 0.140). **Conclusions:** Both perceptions were associated with lower distress. The exploratory interaction explained little additional variance and does not establish a mechanism, clinical benefit, or absence of age differences.

## 1. Introduction

Psychological distress comprises nonspecific symptoms such as depressed mood, nervousness, hopelessness, and worthlessness. The Kessler Psychological Distress Scale (K6) provides a brief population measure of these symptoms [1,2]. National Japanese survey findings describe the distribution of distress and changes over time [3]. Social relationships are one relevant context for understanding distress, but perceptions of being supported and contributing to others are not interchangeable.

Perceived social support concerns the availability or experience of supportive relationships [4]. It differs from reported receipt of supportive behaviors: their association is moderate rather than complete [5]. A systematic review with meta-analyses found associations between perceived support and better mental health [6], and longitudinal Japanese evidence links perceived support to lower K6 scores among middle-aged and young-old adults [7]. The present survey item captures a general feeling of being supported, rather than a specific type or transaction of support.

Perceived usefulness is related to, but narrower than, mattering, which encompasses feeling valued and adding value [8,9]. It also resembles the social contribution dimension of social well-being: perceiving oneself as having something of value to offer society [10]. Perceived burdensomeness in the interpersonal theory of suicide concerns beliefs that one is a liability to others [11]. A low usefulness score is not a validated reverse measure of that construct and cannot support an inference about suicide risk. Role-identity and role-accumulation accounts offer a further rationale: social roles can provide meaning, identity, and opportunities to contribute [12]. Perceived usefulness has also been studied as an indicator distinct from actual role performance [13].

Support exchanges provide a rationale for examining these perceptions together. Imbalances in giving and receiving support have been associated with psychological well-being [14,15]. Research among older Japanese adults emphasizes considering both the amount and balance of support, because similar balance can occur at very different levels of exchange [16]. However, giving support is not equivalent to feeling useful, and the two items in this study do not measure reciprocity. A person can feel supported while reporting little usefulness, or useful while reporting little support.

These accounts do not determine a unique sign for an interaction. A compensatory pattern and a pattern in which the inverse association of one perception is stronger at higher values of the other are both plausible. We therefore examined the interaction as exploratory.

Cultural context may influence these perceptions. Ethnographic work with older people in Japan describes ikigai, relationships and meaningful activity, alongside concerns about meiwaku, or causing trouble to others [17]. These observations motivate attention to roles and contribution, but should not be assumed to characterize every Japanese adult. Neither ikigai nor beliefs about burdening others were measured here.

This study examined associations of feeling supported and perceived usefulness with distress among adults aged ≥20 years in a nationwide internet panel. The interaction, nonlinear patterns, and age group comparisons were exploratory. The study and its hypotheses were not preregistered.

## 2. Materials and Methods

### 2.1. Study Design and Data Source

This cross-sectional secondary analysis used individual-level data from the Survey on Satisfaction and Quality of Life, 2025 (SSJDA study 1834), commissioned by the Cabinet Office, Japan [18]. The seventh wave was conducted from 14 to 31 March 2025 among internet panel members aged 15–89 years living in Japan. Recruitment used nonprobability quotas across 47 prefectures, sex, and five broad age groups (15–24, 25–34, 35–44, 45–59, and ≥60 years) [19]. These quotas should not be interpreted as a probability sample or as guaranteeing a population-proportional age distribution. Survey Research Center Co., Ltd. (Tokyo, Japan) conducted the self-administered web survey. The authors submitted the SSJDA data-use application on 13 August 2026; this application was not a formal study preregistration.

The source survey obtained 10,633 responses; 10,626 records were supplied after source data cleaning [18,19]. The secondary analysis included 10,199 respondents aged ≥20 years after excluding 427 aged 15–19 years. Grouped age data did not allow 18–19-year-olds to be separated from younger respondents. No values were missing in the primary analysis variables. The sample size was determined by the available survey and eligibility criteria, rather than by a new a priori power calculation. Source cleaning and attention-check criteria were not available in the files analyzed.

### 2.2. Feeling Supported and Perceived Usefulness

Feeling supported was measured by an item concerning whether respondents felt that their lives were supported by various people. The usefulness item concerned being useful to people around them or to society. Both were recoded from 11 response levels to 0–10, with higher values indicating stronger endorsement [19]. All respondents answered the Japanese questionnaire; the English item descriptions are translations for reporting. The usefulness item combines interpersonal and societal contribution, which cannot be separated in these data. We examined correlations with other social indicators as descriptive evidence of convergence, without treating them as validation of distinct constructs. Measurement invariance across age groups and the validity of the reporting translations were not assessed.

### 2.3. Psychological Distress

The outcome was a six-item distress score based on K6 item content [1]. The standard Japanese K6 has demonstrated screening validity [20], but this does not validate a modified reference period. The source survey asked about recent life rather than specifying the standard past 30 days [19]. Items concerned depressed mood, nervousness, hopelessness, restlessness, effort, and worthlessness. We use the term K6-derived distress score and do not claim equivalence to the validated K6.

Responses used five frequency categories: all of the time, most of the time, some of the time, a little of the time, and none of the time [19]. Items were scored from 0 (none of the time) to 4 (all of the time) and summed to yield a total of 0–24, with higher scores indicating greater distress. Internal consistency was assessed using Cronbach’s alpha. The total score was analyzed without applying a diagnostic or screening cutoff.

### 2.4. Covariates

The primary analysis adjusted for age, sex, educational attainment, annual household income, living alone, and employment status. Covariates were derived from the source survey [19]. Age was entered using the 13 age categories represented in the adult analytic sample. Sex, educational attainment, household income, and employment status were entered as categorical variables with two, six, seven, and nine categories, respectively. Respondents in one-person households were classified as living alone, whereas those in households with two or more people were classified as not living alone.

Health status was omitted from the primary specification because its cross-sectional relation to distress may involve confounding, mediation, or overlapping appraisal; these data cannot identify which. We therefore report the primary and health-adjusted models side by side. An additional exploratory model included marital status, co-resident children, frequency of contact with friends, the number of people to rely on, loneliness, lack of companions, and major illness or injury during the preceding year. Co-resident children do not identify all respondents who have children. Mental health treatment history was not available in the dataset analyzed. Loneliness and health may overlap with the outcome or lie on a causal pathway, so the extended model is not a definitive confounder-adjusted causal estimate.

### 2.5. Statistical Analysis

Continuous variables were summarized using means, SDs, medians, and IQRs; categorical variables using counts and percentages. Pearson correlations described the focal variables. Spearman correlations related each social perception to additional social indicators. To retain the original parameterization, the social predictors were centered at their means in the supplied dataset of 10,626 respondents: 6.079 for support and 5.011 for usefulness. Centering does not change fitted values or the interaction coefficient.

Ordinary least squares regression modeled the distress score using linear and quadratic terms for both social perceptions and their product, retaining all lower-order terms. This second-order polynomial describes a response surface. Linear coefficients are conditional slopes at the centering values; the product coefficient quantifies interaction on the additive score scale. The model does not assume these items define congruent roles or an agreement construct.

Inference used HC3 heteroskedasticity-robust standard errors [21]. We report unstandardized coefficients, HC3 SEs, normal-approximation 95% confidence intervals, and two-sided *p*-values, together with R^2^, adjusted R^2^, and the increment in R^2^ from adding the product term. HC3 addresses heteroskedasticity in inference; it does not correct an inappropriate conditional mean function or make a bounded outcome unbounded.

Diagnostics examined residuals versus fitted values, residual quantiles, leverage, Cook’s distance, and numerical conditioning. We compared estimates after excluding observations with Cook’s distance >4/n. We counted identical responses across the six distress items, with a separate sensitivity exclusion for identical non-extreme item scores (1–3). Identical responses may be genuine, so these records were retained in the primary analysis. Timing and attention-check data were not available.

Adjusted predictions were used to interpret the interaction. Feeling supported was varied from 3 to 9, approximately its 10th–90th percentile range. Perceived usefulness was set to 4, 5, and 7, corresponding to its first quartile, median, and third quartile. Predictions for each combination were averaged over the observed covariate distribution of the analysis sample. Pointwise 95% confidence intervals for these adjusted mean predictions were calculated using the HC3 covariance matrix and a normal approximation, conditional on the observed covariate distribution.

Sensitivity analyses added health status and the further available social and illness covariates, restricted support to 3–9 and usefulness to 2–8, removed the worthlessness item, and included respondents aged 15–19 years. We also modeled log(1 + distress) to assess outcome-scale dependence. Restricted cubic splines with four knots at the 5th, 35th, 65th, and 95th percentiles modeled each predictor; one model retained a bilinear product, and another used all nine products of their three spline basis functions, tested jointly with an HC3 Wald test. These analyses assess different mean specifications and do not estimate identical interaction parameters.

An exploratory subgroup analysis was conducted among respondents aged ≥65 years. A three-way interaction between age group (<65 versus ≥65 years), feeling supported, and perceived usefulness was tested to assess whether the two-predictor interaction differed by age group. This model allowed the linear and quadratic terms for both social perceptions to vary by age group.

All tests were two-sided; *p*-values are descriptive in this exploratory analysis and were not adjusted for multiple comparisons. All analyses reported in this revision were performed in R version 4.6.1 (R Foundation for Statistical Computing, Vienna, Austria), using base R functions and an explicit HC3 covariance estimator with normal-approximation confidence intervals and two-sided *p*-values. No survey-weight variable was supplied in the analyzed dataset; analyses are unweighted. The study was not preregistered. Reporting is indexed in the accompanying STROBE checklist.

ChatGPT (GPT-6.1 Sol), including Codex assistance (OpenAI, San Francisco, CA, USA), was used during manuscript preparation and revision to assist with drafting and editing text, developing and refining analysis and plotting code, and considering analytical approaches and interpretations. The final analyses were executed by the first author in R (version 4.6.1.; R Foundation for Statistical Computing, Vienna, Austria), and their outputs were checked against the estimates and tables reported in the manuscript. The authors retain responsibility for the analytical choices, interpretations, and final content.

### 2.6. Ethical Considerations

This study used existing microdata from the 2025 Survey on Satisfaction and Quality of Life, provided by SSJDA (study 1834) [18]. The Cabinet Office survey has been conducted since 2019, and earlier waves have been used in published academic research [18,22]. The 2025 microdata are available for secondary analysis through application and approval procedures [18,23]. Chapter 1, Section 3(1)(c)(i) of the Japanese Ethical Guidelines for Medical and Biological Research Involving Human Subjects excludes from its scope studies using only materials or information with established academic value, broad research use, and general availability [24]. The official guidance explains that general availability can include information obtainable by researchers on request from the provider [25]. On the basis of the survey series’ academic use and the documented data-access procedures, the authors assessed this secondary analysis as falling outside the scope of those Guidelines under that provision; accordingly, separate ethical review was not sought. The authors did not recruit participants, collect new data, or access identifying information. SSJDA states that it provides microdata in a form that does not allow identification of individual respondents, and its conditions prohibit identification attempts [26,27].

## 3. Results

### 3.1. Participants and Descriptive Statistics

The primary sample comprised 10,199 adults, including 5134 women (50.3%), 2203 people living alone (21.6%), and 1702 aged ≥65 years (16.7%). Mean support was 6.05 (SD 2.33; median 6, IQR 5–8), and mean usefulness was 4.99 (SD 2.35; median 5, IQR 4–7). Table 1 summarizes the focal measures; Table S1 gives every primary covariate category and health status.

Adults aged ≥65 years represented 16.7% of the sample, compared with 32.9% among those aged 20–89 years in October 2024 population estimates; the broader ≥20-year population figure was 34.7% [28]. Table S2 compares the 13 achieved age categories with the aligned 20–89-year benchmark. This comparison does not establish adherence to the source survey’s broader quota cells.

Mean K6-derived distress was 7.15 (SD 6.47; median 6, IQR 1–12); 2162 respondents (21.2%) scored zero. Cronbach’s α was 0.943. Support and usefulness correlated positively (r = 0.593), and distress correlated negatively with support (r = −0.397) and usefulness (r = −0.465). High internal consistency does not establish validity or absence of item redundancy or response-style effects.

Support/usefulness Spearman correlations were 0.569/0.619 with satisfaction with social connections, −0.377/−0.416 with loneliness, 0.333/0.329 with people to rely on, and 0.272/0.313 with contact frequency. Usefulness correlated 0.820 with feeling recognized. Table S9 reports all eight social indicators. These same-questionnaire correlations are consistent with convergence but also leave shared content and method effects unresolved.

### 3.2. Associations with Psychological Distress

In the primary adjusted model, feeling supported (B = −0.552, 95% CI −0.620 to −0.484) and perceived usefulness (B = −0.744, 95% CI −0.816 to −0.673) were inversely associated with distress near their centering values. Quadratic coefficients were positive for support (B = 0.049, 95% CI 0.024 to 0.074) and usefulness (B = 0.107, 95% CI 0.085 to 0.129; Table 2). These coefficients do not establish a uniformly decreasing association across all score combinations.

The interaction was negative (B = −0.050, 95% CI −0.081 to −0.019, *p* = 0.002). R^2^ was 0.325 and adjusted R^2^ was 0.322. Adding the interaction increased R^2^ by 0.0011, or 0.11 percentage points. Unadjusted and health-adjusted focal coefficients are presented with the primary coefficients in Table S3; Tables S4 and S5 report every primary and health-adjusted coefficient.

### 3.3. Adjusted Predictions

**Figure 1:**
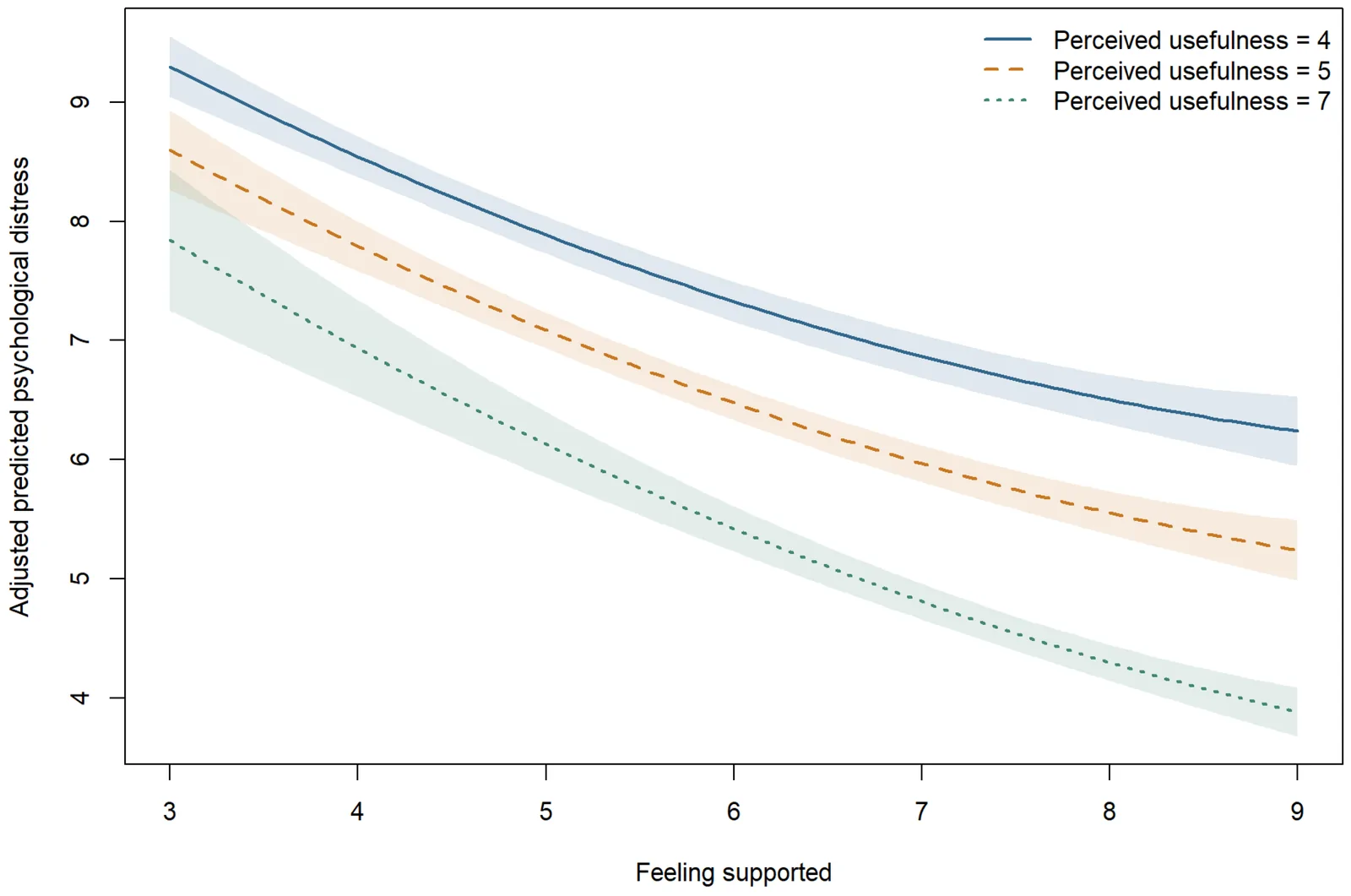
Adjusted predicted psychological distress across feeling-supported scores at selected levels of perceived usefulness. Predictions are from the primary linear regression model with mean-centered linear terms, quadratic terms, and their interaction, adjusted for age, sex, educational attainment, annual household income, living alone, and employment status. They were averaged over the observed covariate distribution of adults aged ≥20 years (*n* = 10,199). Feeling supported is shown from 3 to 9, approximately its 10th–90th percentile range; perceived usefulness is set to 4, 5, and 7 (first quartile, median, and third quartile). The outcome is the K6-derived distress score (0–24), with higher values indicating greater distress. Shaded bands indicate pointwise 95% confidence intervals for adjusted mean predictions, calculated using the HC3 covariance matrix conditional on the observed covariate distribution.

Adjusted predictions averaged over the sample covariate distribution were 9.30 for feeling supported = 3 and perceived usefulness = 4, 6.48 for feeling supported = 6 and perceived usefulness = 5, and 3.89 for feeling supported = 9 and perceived usefulness = 7 (Figure 1).

The predicted difference for support 9 versus 3 was −3.05 points (95% CI −3.43 to −2.68; −0.47 outcome SD) at usefulness 4 and −3.95 (95% CI −4.62 to −3.29; −0.61 SD) at usefulness 7. The difference between these contrasts was −0.90 (95% CI −1.46 to −0.34), equivalent to −0.14 SD (95% CI −0.23 to −0.05). Usefulness 7 versus 4 corresponded to −1.46 points at support 3 and −2.36 at support 9. Table S6 gives all contrast CIs and SD units. These are between-score comparisons, not changes over time. Figure S1 and Table S7 describe the joint surface.

### 3.4. Sensitivity Analyses

Adding health status reduced the linear coefficients to −0.467 for support and −0.553 for usefulness. The interaction was −0.044 (95% CI −0.072 to −0.016, *p* = 0.002). Adjusted R^2^ increased from 0.322 to 0.369, a material change in model fit. With the further available social and illness covariates, the interaction attenuated to −0.025 (95% CI −0.048 to −0.003, *p* = 0.029). This attenuation indicates covariate dependence and should not be described as an unchanged effect size (Table S8).

For log(1 + distress), the interaction was −0.0124 (95% CI −0.0170 to −0.0077). In the spline model retaining a bilinear product, it was −0.046 (95% CI −0.077 to −0.015, *p* = 0.004). The nine spline interaction terms were jointly associated with distress (Wald χ^2^ = 27.38, df = 9, *p* = 0.001). These results support examining a joint pattern under alternative specifications, but coefficients on transformed scales are not directly comparable and do not establish a scale-independent interaction.

In the central-range sample (*n* = 7944), the interaction was −0.113 (95% CI −0.173 to −0.053). Its magnitude was more than twice the primary estimate. This is compatible with flattening or different patterns at extreme combinations, but range restriction also changes the sample and covariate distribution; it does not by itself identify a universal saturation point.

Removing the worthlessness item gave an interaction of −0.040 (95% CI −0.067 to −0.013, *p* = 0.004). This does not exclude overlap involving hopelessness, effort, or general negative appraisal. In all 10,626 supplied respondents, the interaction was −0.046 (95% CI −0.077 to −0.015, *p* = 0.004). Table S8 summarizes the sensitivity analyses.

Focal-term VIFs ranged from 1.95 to 3.02; the centered, scaled design condition index was 9.39. Residual variability depended on fitted values, and 42 fitted scores were below zero. Excluding 548 observations flagged by Cook’s distance yielded an interaction of −0.055; excluding 1082 uniform non-extreme response profiles yielded −0.045. Tables S8 and S10 and Figure S2 report these exploratory diagnostics; neither exclusion defines a superior primary sample.

### 3.5. Exploratory Analyses by Age Group

Among 1702 respondents aged ≥65 years, support (B = −0.408, 95% CI −0.560 to −0.256) and usefulness (B = −0.510, 95% CI −0.675 to −0.344) were inversely associated with distress. The interaction estimate was close to zero but imprecise (B = −0.002, 95% CI −0.077 to 0.073, *p* = 0.959).

The age group difference in the interaction was B = 0.059 (95% CI −0.023 to 0.140, *p* = 0.158). This interval does not establish equivalence or absence of an age-related difference. Full exploratory age-model coefficients are included in the accompanying aggregate analysis output.

## 4. Discussion

### 4.1. Principal Findings

In this cross-sectional general-population internet sample, support and usefulness were inversely associated with distress near their centering values. The additive-score interaction was negative but accounted for only 0.11 percentage points of additional variance. Its magnitude changed with predictor range and covariate adjustment. The main contribution is descriptive: examining two related social perceptions together with nonlinear patterns, rather than establishing a new mechanism or clinically important interaction.

The support association is consistent with prior perceived-support research [6,7]. The usefulness association can be interpreted in relation to mattering, social contribution, and social roles [8,10,12]. However, the items do not identify support sources, actual contributions, or specific roles, and cannot establish that these theoretical constructs explain the observed associations.

The convergence correlations provide useful descriptive context but not discriminant validity. In particular, the usefulness item correlated strongly with feeling recognized (ρ = 0.820). This may indicate shared content or common appraisal as well as a meaningful social perception. The acceptable numerical conditioning of the regression design does not establish that the items measure separable latent constructs.

### 4.2. Joint and Nonlinear Associations

The −5.41-point contrast between support/usefulness scores of 9/7 and 3/4 reflects both predictors and their curvature, not the interaction alone. The interaction-specific difference in contrasts was −0.90 points, or −0.14 SD. These conditional comparisons quantify the surface within selected ranges; no minimum clinically important difference has been established for the modified outcome. Statistical significance should not be interpreted as a clinical benefit.

The larger central-range interaction is compatible with flattening at the extremes, but also reflects a selected sample. Spline results make the quadratic specification less decisive: a joint pattern remained, without requiring that one second-order polynomial describe all combinations. The log-transformed outcome also retained a negative product coefficient, but additive and log-scale interactions have different meanings. Thus, the sign was similar across the reported checks, while effect magnitude and interpretation were specification-dependent.

### 4.3. Social Relationships and Roles

Relational regulation theory links perceived support to affect, thought, and action through ordinary conversation and shared activities [29]. Role-based meaning, self-esteem, and perceived control are other proposed pathways linking social ties to health [12,30]. These perspectives provide concise interpretations and questions for future research. None of these processes, perceived burdensomeness, or ikigai were directly tested here.

### 4.4. Age Comparisons and Future Research

The older subgroup estimate and the direct age group comparison had wide confidence intervals. Its interaction SE was about 2.4 times the full-sample SE, compatible with the roughly 2.4-fold difference expected from the sample-size ratio; this does not establish that sample size is the only source of uncertainty. Absence of statistical significance does not establish absence of an association or equivalence between groups [31].

These findings concern general-population social perceptions. They do not demonstrate relevance to a nursing process or effectiveness of a clinical intervention. Studies in care settings could examine whether experiences of receiving support and having opportunities to contribute are associated with distress, using validated measures and longitudinal designs. Clinical recommendations require evidence beyond this survey.

### 4.5. Strengths

A strength of this study is its large sample of 10,199 adults.

### 4.6. Limitations

First, cross-sectional associations cannot establish temporal ordering: distress may alter perceptions of support and usefulness, and unmeasured factors may affect all three. The attenuation in the extended model is consistent with shared variation but cannot distinguish confounding from mediation or overlapping measurement. Second, the unweighted nonprobability internet sample underrepresented older adults compared with population estimates. Age covariates do not make the sample representative, and subgroup precision and generalization remain limited.

Third, single items cannot distinguish support sources, quality, behaviors, or interpersonal from societal usefulness. The usefulness item is double-barreled. Convergent correlations and VIFs do not validate distinct constructs. Age-related measurement invariance was not evaluated; subgroup differences may reflect differences in item interpretation. Multidimensional measures, such as the 2-Way Social Support Scale [32], would permit more detailed assessment.

Fourth, shared questionnaire method, general negative appraisal, and response styles may contribute to the associations [33]. Removing worthlessness does not remove overlap involving hopelessness or effort. Identical responses across distress items were common, but may reflect real symptom profiles and cannot be labeled inattentive without additional evidence. Attention-check and completion-time information was not available for this secondary analysis. The uniform-response exclusion is therefore a sensitivity check, not a validated quality filter.

Fifth, the modified reference period limits comparability with validated K6 data and screening cutoffs. The observed mean of 7.15 is high relative to standard-K6 Japanese population reports. Descriptively, 38.7% scored 5–12 and 18.7% scored 13–24, compared with approximately 21.8% and 5.0% in a published national 2022 analysis [3]. These are not comparable diagnostic prevalence estimates: reference period, survey year, age coverage, weighting, and sampling differ. Web-panel selection and the unspecified recent-life period are plausible explanations that cannot be separated here. Alpha indicates consistency, not validity. Finally, residual heteroskedasticity and 42 fitted values below zero show limits of OLS for this bounded score; HC3 and alternative models do not make it an individual clinical prediction tool.

## 5. Conclusions

Feeling supported and perceived usefulness were each associated with lower distress in this Japanese internet sample. An exploratory interaction added little explanatory variance and varied in magnitude across specifications. These findings highlight the need for a longitudinal study of the two social perceptions with validated measures and more representative samples. They do not establish a mechanism, clinical benefit, or absence of age differences.

## Figures and Tables

**Table 1:** Selected characteristics of adults aged ≥20 years (*n* = 10,199).

Characteristic	Value
Women, *n* (%)	5134 (50.3)
Living alone, *n* (%)	2203 (21.6)
Feeling supported, mean (SD)	6.05 (2.33)
Feeling supported, median (IQR)	6 (5–8)
Perceived usefulness, mean (SD)	4.99 (2.35)
Perceived usefulness, median (IQR)	5 (4–7)
Psychological distress, mean (SD)	7.15 (6.47)
Psychological distress, median (IQR)	6 (1–12)
Psychological distress score of zero, %	21.2
Aged ≥65 years, *n* (%)	1702 (16.7)

SD, standard deviation; IQR, interquartile range. Social perceptions range from 0 to 10; the K6-derived distress score ranges from 0 to 24 and uses the source survey’s modified reference period. Full categorical distributions are in Table S1.

**Table 2:** Associations of feeling supported and perceived usefulness with psychological distress in the primary adjusted model (*n* = 10,199).

Model Term	B	HC3 SE	95% CI	*p*
Feeling supported	−0.552	0.035	−0.620 to −0.484	<0.001
Feeling supported squared	0.049	0.013	0.024 to 0.074	<0.001
Perceived usefulness	−0.744	0.037	−0.816 to −0.673	<0.001
Perceived usefulness squared	0.107	0.011	0.085 to 0.129	<0.001
Feeling supported × perceived usefulness	−0.050	0.016	−0.081 to −0.019	0.002

B, unstandardized coefficient; HC3 SE, heteroskedasticity-robust standard error; CI, confidence interval. Models adjust for age, sex, education, household income, living alone, and employment. Support and usefulness are centered at 6.079 and 5.011. Linear terms are conditional slopes at these values. R^2^ = 0.325; adjusted R^2^ = 0.322; interaction ΔR^2^ = 0.0011. Full coefficients and reference categories are in Tables S4 and S5.

## Data Availability

The data analyzed in this study are not publicly available from the authors because the individual-level data were provided under the terms of use of the Social Science Japan Data Archive and may not be redistributed to unauthorized third parties. The data are available from the Social Science Japan Data Archive, Center for Social Research and Data Archives, Institute of Social Science, The University of Tokyo, upon application and approval (SSJDA study number 1834; https://doi.org/10.34500/SSJDA.1834). The R code used to reproduce the revised analyses and figures is provided with the Supplementary Material, together with aggregate analysis outputs and R session information. Individual-level data are not included in those files.

## Supplementary Materials

The following supporting information can be downloaded at: https://www.mdpi.com/article/10.3390/healthcare14203364/s1, Table S1. Complete sample characteristics. Table S2. Achieved age distribution and population benchmark. Table S3. Focal coefficients across adjustment sets. Table S4. Full primary model. Table S5. Full health-adjusted model. Table S6. Adjusted score contrasts and outcome SD units. Table S7. Descriptive joint response surface. Table S8. Sensitivity and exploratory interaction estimates. Table S9. Correlations with additional social indicators. Table S10. Numerical and data diagnostics. Figure S1. Descriptive joint response surface. Figure S2. Numerical and data diagnostics.

## Abbreviations

HC3	Heteroskedasticity-consistent covariance matrix estimator, type 3
IQR	Interquartile range
K6	Kessler 6-item Psychological Distress Scale
SD	Standard deviation
SE	Standard error
SSJDA	Social Science Japan Data Archive
