# Supplementary material for: Feeling Supported, Perceived Usefulness, and Psychological Distress Among Adults in Japan: A Cross-Sectional Study
Source: Healthcare (Basel). 2026 Oct 9;14(20):3364. doi: 10.3390/healthcare14203364 (PMC13704007; doi:10.3390/healthcare14203364)
Supplement: Supplementary file 1 [file healthcare-14-03364-s001.zip › healthcare-4600146-supplementary.pdf]

Supplementary material

Feeling Supported, Perceived Usefulness, and Psychological Distress among Adults in Japan: A Cross-Sectional Study
Shimpei Hayashi and Shigeaki Masuda

Table S1. Complete sample characteristics

Adults aged ≥20 years; n = 10,199. All primary categorical covariates and the health sensitivity covariate are shown. Percentages use the same denominator.

Characteristic	Category	n	%
Age	20–24 years	1,458	14.3
Age	25–29 years	861	8.4
Age	30–34 years	1,329	13.0
Age	35–39 years	926	9.1
Age	40–44 years	1,151	11.3
Age	45–49 years	733	7.2
Age	50–54 years	748	7.3
Age	55–59 years	686	6.7
Age	60–64 years	605	5.9
Age	65–69 years	761	7.5
Age	70–74 years	547	5.4
Age	75–79 years	301	3.0
Age	80–89 years	93	0.9
Sex	Male	5,065	49.7
Sex	Female	5,134	50.3
Education	Junior high school	236	2.3
Education	High school	2,791	27.4
Education	Vocational school	1,273	12.5
Education	Technical college / junior college	1,065	10.4
Education	University	4,363	42.8
Education	Graduate school	471	4.6
Household income	<JPY 1 million	784	7.7
Household income	JPY 1 to <3 million	1,868	18.3

Characteristic	Category	n	%
Household income	JPY 3 to <5 million	2,663	26.1
Household income	JPY 5 to <7 million	2,031	19.9
Household income	JPY 7 to <10 million	1,751	17.2
Household income	JPY 10 to <20 million	960	9.4
Household income	≥JPY 20 million	142	1.4
Living arrangement	Not living alone	7,996	78.4
Living arrangement	Living alone	2,203	21.6
Employment	Regular employee	4,468	43.8
Employment	Nonregular employee	1,922	18.8
Employment	Company executive	183	1.8
Employment	Self-employed	596	5.8
Employment	Family worker / home piecework	185	1.8
Employment	Student with part-time work	431	4.2
Employment	Student without part-time work	137	1.3
Employment	Nonstudent, unemployed and seeking work	286	2.8
Employment	Nonstudent, not seeking work	1,991	19.5
Health status	Good	1,330	13.0
Health status	Somewhat good	3,006	29.5
Health status	Average	3,516	34.5
Health status	Not very good	1,817	17.8
Health status	Poor	530	5.2

Education categories describe completed or expected attainment. The archived top age category is ≥80 years; the source survey's eligible range ends at 89 years. Income is annual household income. Labels are in English, reporting translations of the Japanese source categories.

Table S2. Achieved age distribution and population benchmark

Age	Sample n	Sample %	Population, thousands	Population %
20–24 years	1,458	14.3	6,261	6.2
25–29 years	861	8.4	6,518	6.4
30–34 years	1,329	13.0	6,396	6.3
35–39 years	926	9.1	6,872	6.8
40–44 years	1,151	11.3	7,631	7.5
45–49 years	733	7.2	8,747	8.6
50–54 years	748	7.3	9,788	9.6
55–59 years	686	6.7	8,490	8.3
60–64 years	605	5.9	7,573	7.4
65–69 years	761	7.5	7,268	7.1
70–74 years	547	5.4	8,198	8.1
75–79 years	301	3.0	7,886	7.8
80–89 years	93	0.9	10,082	9.9
65–89 years	1,702	16.7	33,434	32.9

Benchmark: Statistics Bureau of Japan, Population Estimates as of 1 October 2024, Table 1 (total population, both sexes), aligned to ages 20–89 years. Single-year rounded counts were summed to the 13 survey categories; denominator 101,710 thousand. Small differences from separately rounded published totals arise from rounding. The share aged ≥65 years in the broader ≥20-year population was 34.7%; the aligned share above is 32.9%. This is a composition benchmark, not a test of the source survey's 47-prefecture × sex × five-broad-age quota cells.

Source: https://www.stat.go.jp/data/jinsui/2024np/pdf/2024tables.pdf. Source survey quotas: https://www5.cao.go.jp/keizai2/wellbeing/manzoku/chousahyou7_1.pdf.

Table S3. Focal coefficients across adjustment sets

Entries are B (95% HC3 CI), on the original 0–24 distress scale. Every model includes both linear terms, both quadratic terms, and their product. Unadjusted means no additional demographic covariates; it is not a series of separate single-predictor regressions.

Term	Unadjusted	Primary adjusted	+ Health status
Support	−0.595 (−0.664 to −0.525)	−0.552 (−0.620 to −0.484)	−0.467 (−0.532 to −0.403)
Support squared	0.062 (0.035 to 0.088)	0.049 (0.024 to 0.074)	0.040 (0.016 to 0.063)
Usefulness	−0.775 (−0.849 to −0.702)	−0.744 (−0.816 to −0.673)	−0.553 (−0.623 to −0.484)
Usefulness squared	0.129 (0.106 to 0.152)	0.107 (0.085 to 0.129)	0.098 (0.077 to 0.119)
Support × usefulness	−0.069 (−0.102 to −0.036)	−0.050 (−0.081 to −0.019)	−0.044 (−0.072 to −0.016)

Primary adjustment: age, sex, education, household income, employment, and living alone. Health adjustment adds four indicators for the five-category health variable. Primary R^2/adjusted R^2 = 0.325/0.322; health-adjusted = 0.372/0.369. The improvement in fit is material and does not identify whether health is a confounder, mediator, or overlapping appraisal.

Table S4. Full primary model

n = 10,199. All coefficients, including the intercept and categorical covariates, are shown. Support/usefulness are centered at 6.079/5.011. Reference categories: age 20–24; male; junior high school; income <JPY 1 million; regular employee; not living alone.

Term	B	HC3 SE	95% CI	p
Intercept	9.289	0.543	8.225 to 10.353	<0.001
Support	−0.552	0.035	−0.620 to −0.484	<0.001
Support squared	0.049	0.013	0.024 to 0.074	<0.001
Usefulness	−0.744	0.037	−0.816 to −0.673	<0.001
Usefulness squared	0.107	0.011	0.085 to 0.129	<0.001
Support × usefulness	−0.050	0.016	−0.081 to −0.019	0.002
Age: 25–29 years	−0.712	0.270	−1.241 to −0.183	0.008
Age: 30–34 years	−1.280	0.246	−1.763 to −0.797	<0.001
Age: 35–39 years	−1.194	0.267	−1.718 to −0.671	<0.001
Age: 40–44 years	−1.625	0.252	−2.120 to −1.131	<0.001
Age: 45–49 years	−2.069	0.285	−2.628 to −1.511	<0.001
Age: 50–54 years	−2.416	0.276	−2.957 to −1.875	<0.001
Age: 55–59 years	−2.904	0.283	−3.459 to −2.350	<0.001

Term	B	HC3 SE	95% CI	p
Age: 60–64 years	−3.842	0.286	−4.402 to −3.282	<0.001
Age: 65–69 years	−4.832	0.267	−5.356 to −4.308	<0.001
Age: 70–74 years	−5.390	0.288	−5.954 to −4.827	<0.001
Age: 75–79 years	−5.465	0.315	−6.083 to −4.847	<0.001
Age: 80–89 years	−4.367	0.482	−5.312 to −3.421	<0.001
Sex: Female	−0.297	0.117	−0.526 to −0.069	0.011
Education: High school	−0.273	0.445	−1.145 to 0.600	0.540
Education: Vocational school	−0.092	0.458	−0.991 to 0.807	0.841
Education: Technical college / junior college	−0.019	0.463	−0.926 to 0.887	0.967
Education: University	−0.051	0.442	−0.918 to 0.816	0.908
Education: Graduate school	−0.311	0.498	−1.287 to 0.664	0.532
Household income: JPY 1 to <3 million	−0.047	0.258	−0.554 to 0.459	0.854
Household income: JPY 3 to <5 million	−0.254	0.252	−0.748 to 0.239	0.312
Household income: JPY 5 to <7 million	−0.610	0.262	−1.123 to −0.097	0.020
Household income: JPY 7 to <10 million	−0.988	0.271	−1.519 to −0.458	<0.001
Household income: JPY 10 to <20 million	−0.916	0.295	−1.494 to −0.338	0.002
Household income: ≥JPY 20 million	−0.486	0.550	−1.564 to 0.593	0.377
Employment: Nonregular employee	0.175	0.165	−0.149 to 0.499	0.290
Employment: Company executive	1.408	0.395	0.634 to 2.183	<0.001
Employment: Self-employed	−0.241	0.235	−0.702 to 0.220	0.306
Employment: Family worker / home piecework	0.709	0.439	−0.152 to 1.570	0.106
Employment: Student with part-time work	−0.784	0.325	−1.422 to −0.147	0.016
Employment: Student without part-time work	−0.787	0.498	−1.764 to 0.190	0.114
Employment: Nonstudent, unemployed and seeking work	0.432	0.361	−0.276 to 1.140	0.232
Employment: Nonstudent, not seeking work	−0.248	0.195	−0.629 to 0.134	0.203
Living alone (ref: not alone)	0.053	0.146	−0.233 to 0.340	0.715

CI and p-values use an HC3 covariance matrix and a normal approximation. Covariate coefficients are adjustment parameters rather than prespecified causal effects.

Table S5. Full health-adjusted model

n = 10,199. All coefficients, including the intercept and categorical covariates, are shown. Support/usefulness are centered at 6.079/5.011. Reference categories: age 20–24; male; junior high school; income <JPY 1 million; regular employee; not living alone; good health.

Term	B	HC3 SE	95% CI	p
Intercept	7.062	0.550	5.983 to 8.140	<0.001
Support	−0.467	0.033	−0.532 to −0.403	<0.001
Support squared	0.040	0.012	0.016 to 0.063	<0.001
Usefulness	−0.553	0.035	−0.623 to −0.484	<0.001
Usefulness squared	0.098	0.011	0.077 to 0.119	<0.001
Support × usefulness	−0.044	0.014	−0.072 to −0.016	0.002
Age: 25–29 years	−0.750	0.263	−1.265 to −0.235	0.004
Age: 30–34 years	−1.300	0.237	−1.764 to −0.835	<0.001
Age: 35–39 years	−1.345	0.258	−1.850 to −0.840	<0.001
Age: 40–44 years	−1.843	0.242	−2.317 to −1.368	<0.001
Age: 45–49 years	−2.341	0.275	−2.880 to −1.802	<0.001
Age: 50–54 years	−2.728	0.266	−3.249 to −2.207	<0.001
Age: 55–59 years	−3.270	0.274	−3.806 to −2.733	<0.001
Age: 60–64 years	−4.109	0.277	−4.652 to −3.566	<0.001
Age: 65–69 years	−5.086	0.260	−5.595 to −4.578	<0.001
Age: 70–74 years	−5.720	0.281	−6.271 to −5.169	<0.001
Age: 75–79 years	−5.796	0.308	−6.401 to −5.191	<0.001
Age: 80–89 years	−4.786	0.476	−5.718 to −3.854	<0.001
Sex: Female	−0.201	0.113	−0.422 to 0.020	0.075
Education: High school	−0.006	0.436	−0.859 to 0.848	0.990
Education: Vocational school	0.142	0.448	−0.737 to 1.020	0.752
Education: Technical college / junior college	0.300	0.452	−0.586 to 1.186	0.507
Education: University	0.250	0.434	−0.600 to 1.100	0.564
Education: Graduate school	0.095	0.488	−0.862 to 1.052	0.845
Household income: JPY 1 to <3 million	−0.069	0.250	−0.560 to 0.422	0.783
Household income: JPY 3 to <5 million	−0.117	0.244	−0.595 to 0.361	0.632
Household income: JPY 5 to <7 million	−0.484	0.255	−0.983 to 0.015	0.057

Term	B	HC3 SE	95% CI	p
Household income: JPY 7 to <10 million	−0.835	0.263	−1.350 to −0.320	0.001
Household income: JPY 10 to <20 million	−0.804	0.287	−1.367 to −0.241	0.005
Household income: ≥JPY 20 million	−0.479	0.528	−1.514 to 0.556	0.364
Employment: Nonregular employee	0.051	0.160	−0.262 to 0.364	0.750
Employment: Company executive	1.271	0.397	0.493 to 2.049	0.001
Employment: Self-employed	−0.355	0.228	−0.802 to 0.093	0.121
Employment: Family worker / home piecework	0.395	0.435	−0.458 to 1.247	0.365
Employment: Student with part-time work	−0.692	0.317	−1.314 to −0.070	0.029
Employment: Student without part-time work	−0.822	0.483	−1.769 to 0.125	0.089
Employment: Nonstudent, unemployed and seeking work	0.102	0.336	−0.557 to 0.760	0.762
Employment: Nonstudent, not seeking work	−0.610	0.186	−0.975 to −0.245	0.001
Health status: Somewhat good	1.298	0.169	0.967 to 1.629	<0.001
Health status: Average	2.170	0.175	1.827 to 2.514	<0.001
Health status: Not very good	3.907	0.201	3.513 to 4.301	<0.001
Health status: Poor	6.747	0.330	6.100 to 7.393	<0.001
Living alone (ref: not alone)	0.065	0.142	−0.213 to 0.343	0.647

CI and p-values use an HC3 covariance matrix and a normal approximation. Covariate coefficients are adjustment parameters rather than prespecified causal effects.

Table S6. Adjusted score contrasts and outcome SD units

Contrast	Score difference (95% CI)	Outcome SD units (95% CI)
Support 9 versus 3 at usefulness 4	−3.05 (−3.43 to −2.68)	−0.47 (−0.53 to −0.41)
Support 9 versus 3 at usefulness 7	−3.95 (−4.62 to −3.29)	−0.61 (−0.71 to −0.51)
Usefulness 7 versus 4 at support 3	−1.46 (−1.92 to −1.00)	−0.22 (−0.30 to −0.15)
Usefulness 7 versus 4 at support 9	−2.36 (−2.61 to −2.10)	−0.36 (−0.40 to −0.32)
Difference in support contrasts	−0.90 (−1.46 to −0.34)	−0.14 (−0.23 to −0.05)
Both high versus both lower	−5.41 (−5.75 to −5.07)	−0.84 (−0.89 to −0.78)

Predictions average over the observed covariate distribution. Outcome SD = 6.4738; SD units divide a score contrast and its CI by this observed SD, treated as fixed. They are descriptive standardized contrasts, not standardized regression coefficients. “Both high versus both lower” compares (support, usefulness) = (9,7) versus (3,4). Clinical importance has not been established.

Support	Usefulness	Adjusted mean	95% CI
3	4	9.30	9.04 to 9.55
3	5	8.60	8.26 to 8.93
3	7	7.84	7.25 to 8.43
6	4	7.33	7.16 to 7.50
6	5	6.48	6.33 to 6.62
6	7	5.42	5.23 to 5.61
9	4	6.24	5.95 to 6.53
9	5	5.24	4.99 to 5.50
9	7	3.89	3.68 to 4.09

Table S7 and Figure S1. Descriptive joint response surface

Surface parameter	Estimate	95% CI	p
Slope along support = usefulness at (5,5)	−1.350	−1.418 to −1.282	<0.001
Curvature along support = usefulness	0.106	0.086 to 0.126	<0.001
Slope along support + usefulness = 10 at (5,5)	0.036	−0.140 to 0.211	0.690
Curvature along support + usefulness = 10	0.206	0.145 to 0.267	<0.001

The lines support = usefulness and support + usefulness = 10 describe directions through (5,5) on the common 0–10 response scale. Because the items have different meanings, these lines do not represent validated congruence or discrepancy constructs. Curvature entries are coefficients of the squared line coordinate, not second derivatives. CIs are linear combinations of the primary coefficients and HC3 covariance matrix.

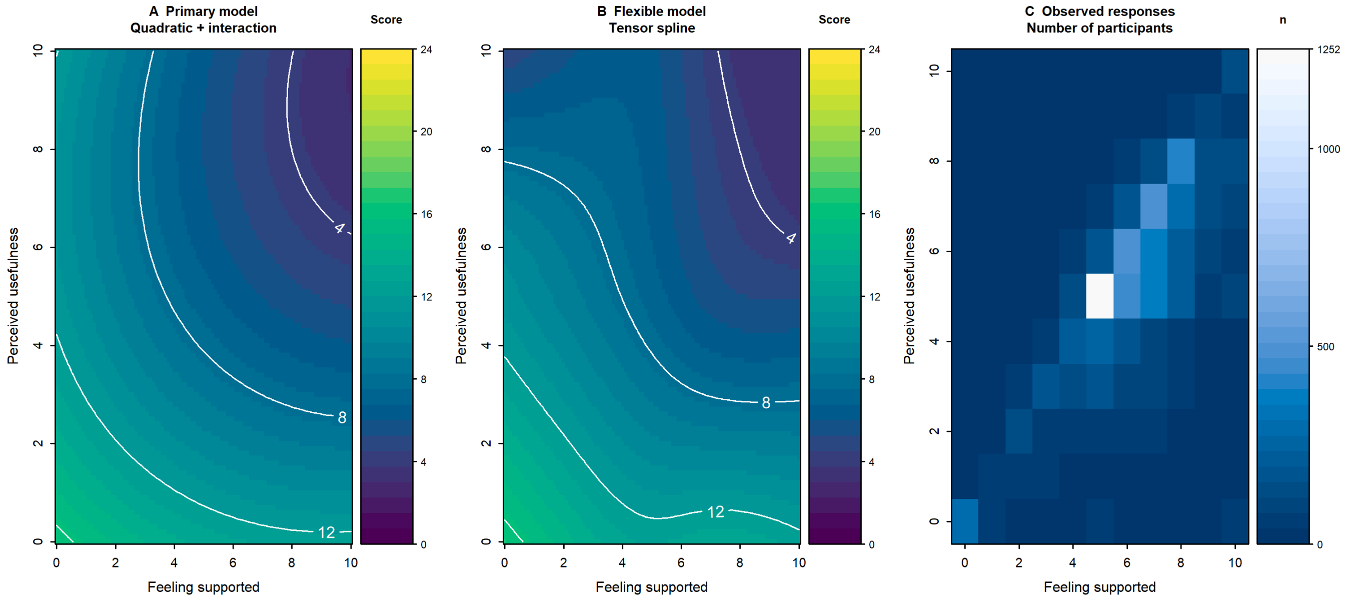

Figure S1. (A) Adjusted mean distress from the primary quadratic model with a support-by-usefulness interaction. (B) Adjusted mean distress from the flexible tensor spline model. Both surfaces average over the sample covariates. Panels A and B use the same color scale, from 0 to 24, with 32 equal-width color bins; purple indicates lower and yellow higher predicted distress. White contours mark distress scores 4, 8, 12, and 16. (C) Observed participant counts for each support/usefulness score combination, using 32 equal-width color bins from 0 to 1,252 participants; darker blue indicates fewer and lighter colors more participants. Color bars identify the values. Sparse combinations and score extremes require caution; fitted surfaces are not individual clinical predictions. Tensor splines use four knots per predictor and nine basis-product terms; the joint test is reported below.

Table S8. Sensitivity and exploratory interaction estimates

Model	n	Interaction B	95% CI	p
Primary additive score model	10,199	−0.050	−0.081 to −0.019	0.002
+ Health status	10,199	−0.044	−0.072 to −0.016	0.002
+ Health and additional social/illness variables	10,199	−0.025	−0.048 to −0.003	0.029
Age ≥65 subgroup	1,702	−0.002	−0.077 to 0.073	0.959
Central predictor ranges	7,944	−0.113	−0.173 to −0.053	<0.001
Without worthlessness (0–20)	10,199	−0.040	−0.067 to −0.013	0.004
All supplied ages (≥15)	10,626	−0.046	−0.077 to −0.015	0.004
Log(1 + distress)	10,199	−0.0124	−0.0170 to −0.0077	<0.001
Spline main effects + bilinear interaction	10,199	−0.046	−0.077 to −0.015	0.004
Cook-flagged observations excluded	9,651	−0.055	−0.079 to −0.031	<0.001
Uniform non-extreme item profiles excluded	9,117	−0.045	−0.077 to −0.013	0.006

The health and extended models have adjusted $R^2 = 0.369$ and 0.654, respectively. Extended adjustment adds marital status (including spouse living apart), co-resident children, categorical friend-contact frequency and people-to-rely-on counts, loneliness, lack of companions, and major illness/injury in the preceding year. These variables may be confounders, mediators, or overlapping constructs; attenuation is not a uniquely causal interpretation.

Central ranges: support 3–9, usefulness 2–8. The more than doubled coefficient reflects a different sample and modeled surface; it is compatible with extreme-range flattening, without proving it. The five-item score can still overlap semantically with usefulness through effort and hopelessness. The transformed-outcome coefficient is on a different scale and is not directly comparable with additive-score coefficients.

Restricted cubic spline knots were support: 2, 5, 7, 10; usefulness: 0, 5, 6, 8. All nine basis-product interaction terms were tested jointly: Wald $\chi^2 = 27.38$, df = 9, $p = 0.0012$. This is a flexible joint association test, not a direct estimate of one bilinear interaction. The three-way age modification in the polynomial model was B = 0.059, HC3 SE = 0.041, 95% CI −0.023 to 0.140, $p = 0.158$. It does not establish equivalence.

Table S9. Correlations with additional social indicators

Indicator	Support ρ	Usefulness ρ
Any community or volunteer activity	0.171	0.224
Contact frequency ordered category	0.272	0.313
Feeling accepted when differing from others	0.591	0.756
Feeling recognized by others	0.622	0.820
Lack of companions frequency	−0.356	−0.392
Loneliness frequency	−0.377	−0.416
Number of people to rely on ordered category	0.333	0.329
Satisfaction with social connections	0.569	0.619

Spearman correlations; $n = 10{,}199$ for every pair, all $p < 0.001$. Larger scores mean more recognition, acceptance, connection satisfaction, people to rely on, or contact, and more loneliness or lack of companions for those two indicators. Community activity is any reported activity (versus none). Correlations describe convergence and shared variation within one questionnaire; they do not establish discriminant validity, age invariance, or independent validation. The usefulness–recognition correlation of 0.820 warrants particular caution about overlapping content.

Table S10 and Figure S2. Numerical and data diagnostics

Diagnostic	Result
Focal-term VIFs	Support 2.09; support² 2.57; usefulness 2.14; usefulness² 1.95; product 3.02
Centered, scaled design condition index	9.39 (largest/smallest singular value; intercept excluded)
Outcome distribution	Mean 7.15; SD 6.47; skewness 0.746; 2,162 zeros (21.2%)
OLS fitted values outside 0–24	42 below 0 (0.4%); none above 24
Cook’s distance	Maximum 0.00767; 548 exceed $4/n = 0.000392$
Leverage	Maximum 0.04146; 792 exceed $2p/n$ ($p = 39$ parameters)
Identical responses on six distress items	3,543 (34.7%), including legitimate zero or maximum profiles
Identical non-extreme item responses	1,082 (10.6%), all six item scores equal 1, 2, or 3
Residual variance association	HC3 Wald test of fitted/fitted² in regression of squared residuals: $\chi^2 = 592.70$, df = 2, $p < 0.001$

Cook and leverage cutoffs flag observations for examination and are not deletion rules for the primary analysis. Neither identical symptom responses nor their exclusion can establish inattentiveness without external quality information. Attention checks, completion times, and the detailed source-cleaning rules were not supplied. Numerical conditioning does not establish construct validity. Nonconstant variance supports robust inference, while the residual pattern and out-of-range fitted values limit the conditional mean model.

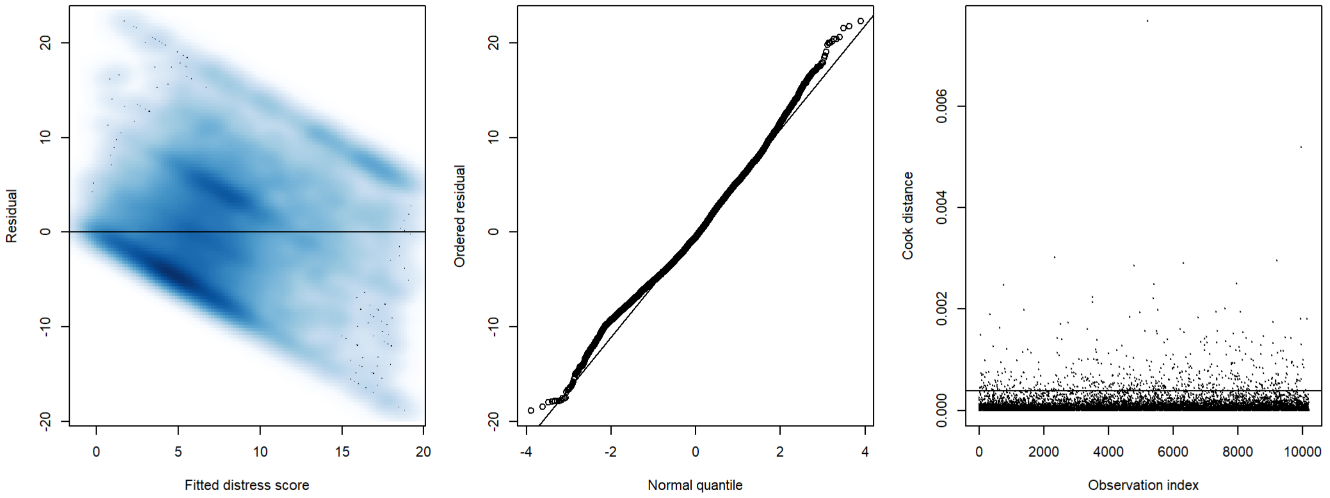

Figure S2. Residuals versus fitted scores, normal-quantile plot, and Cook's distance. The horizontal reference line in the influence panel is 4/n. The normal-quantile plot is descriptive; OLS with HC3 does not require normally distributed individual residuals for its large-sample inference.

Reproducibility and data access

All reported analyses were run in R version 4.6.1. The accompanying analysis folder provides reanalyze_1834_R.R, aggregate results in results_R/, and sessionInfo.txt. The script requires an independently authorized, CP932-encoded copy of SSJDA study 1834. Place it at analysis/source/1834.csv or set the input-path option described in README.txt, and run the script from the package root. A fresh run writes to analysis/results_R_new/ to preserve the supplied results. The study was not preregistered; the SSJDA data-use application was submitted on 13 August 2026. No individual-level survey data are included. Data application and approval: https://doi.org/10.34500/SSJDA.1834. The main script calls update_S1_1834_R.R to produce the final labeled Figure S1; this helper can also redraw S1 from the saved coefficients without refitting models.
